# Exploring effective characteristics of e-cigarette prevention videos among Chinese adolescents: A qualitative focus group study

**DOI:** 10.18332/tid/211477

**Published:** 2025-10-24

**Authors:** Yu Chen, Haoyi Liu, Rui Zhang, Xinjie Zhao, Yujiang Cai, Jing Xu, Kin-Sun Chan

**Affiliations:** 1 School of Art and Communication, Fujian Polytechnic Normal University, Fuzhou, China; 2 Department of Media and Communication, City University of Hong Kong, Hong Kong SAR, China; 3 Faculty of Social Sciences, University of Macau, Macao SAR, China; 4 School of Journalism and Communication, Peking University, Beijing, China; 5 School of International Studies, Peking University, Beijing, China; 6 Institute for Global Health and Development, Peking University, Beijing, China

**Keywords:** e-cigarette prevention, adolescents, focus groups, qualitative research, health communication

## Abstract

**INTRODUCTION:**

The rapid proliferation of electronic cigarettes (e-cigarettes) among adolescents represents a significant public health challenge globally. This qualitative study explored adolescents’ perspectives on effective characteristics of e-cigarette prevention videos to inform evidence-based prevention strategies.

**METHODS:**

We conducted four focus groups with 40 middle-school students aged 13–15 years in 2021 in Beijing and Kunming, China. Using purposive sampling, we selected participants (n=40) from schools in cities with contrasting tobacco control environments. After viewing four international e-cigarette prevention videos varying in style and content, participants discussed perceived effective and ineffective characteristics. We employed Braun and Clarke’s six-phase reflexive thematic analysis approach, using NVivo 12 for data management.

**RESULTS:**

Thematic analysis identified two main domains: effective and ineffective video characteristics. Effective features included authentic case studies demonstrating real consequences, specific health hazards with visual impact, disclosure of harmful chemical components, and appropriately disturbing imagery that created emotional response. Fear appeals and emotional narratives proved particularly impactful when combined with concrete information. Ineffective characteristics included animation formats, overly complex or vague information, didactic expert testimonials, and excessive video length (>2 minutes). Participants recommended that future videos incorporate real-life cases, specific health consequences, moderate fear appeals, and concise messaging within 1–3 minutes.

**CONCLUSIONS:**

Chinese adolescents respond most effectively to prevention videos featuring authentic narratives and specific health consequences rather than animated or didactic content. The preference for fear appeals combined with factual information suggests that emotionally engaging yet informative content may optimize prevention effectiveness. These findings provide evidence for developing culturally appropriate e-cigarette prevention video development for Chinese youth, particularly given ongoing challenges in policy enforcement and youth access to e-cigarettes.

## INTRODUCTION

The rapid proliferation of electronic cigarettes (e-cigarettes) among adolescents represents a significant public health challenge globally^1^. The World Health Organization defines e-cigarettes as electronic nicotine delivery systems (ENDS) and electronic non-nicotine delivery systems (ENNDS) as devices that heat liquid to produce aerosol for inhalation, typically containing nicotine, propylene glycol, glycerin, and flavorings^2^. Despite comprehensive regulatory measures implemented by the Chinese government, including the 2018 prohibition on sales to minors and the 2022 Electronic Cigarette Management Measures, adolescent e-cigarette use remains a challenge^3^.

According to China Disease Prevention and Control Center surveillance data, the prevalence of e-cigarette use among Chinese middle-school students increased significantly between 2019 and 2021, with lifetime use reaching 16.1% and current use at 3.6%, representing increases of 3.5 and 0.8 percentage points, respectively^4^. Recent evidence from Shenzhen indicates that 47.83% of adolescent e-cigarette purchases involved no age verification, and 85% of retail outlets continued stocking banned flavored products in 2023, demonstrating significant enforcement challenges^5^. These implementation gaps underscore the importance of complementary educational interventions to protect youth from e-cigarette initiation.

Video-based health education has emerged as a particularly promising medium for adolescent tobacco prevention. The visual medium’s capacity to combine emotional narrative with factual information aligns well with adolescent information processing preferences and social media consumption patterns^6^. A recent scoping review of e-cigarette prevention messages identified that messages combining health consequences with emotional appeals demonstrated greater effectiveness among adolescents than purely informational content^7^.

However, existing prevention videos have primarily been developed for Western audiences, with limited consideration of cultural factors influencing Chinese adolescents’ reception of prevention messages^6,7^. The majority of research on e-cigarette prevention has focused on older adolescents and young adults, with insufficient attention to the critical age group of 13–15 years when experimentation often begins^8^. Furthermore, qualitative research exploring adolescents’ own perspectives on what makes prevention videos effective remains scarce, particularly in the Chinese context.

Theoretical frameworks from health communication suggest that message framing significantly influences prevention effectiveness. Frame theory, originally developed by Entman^9^ and adapted for health communication research, posits that how information is selected, emphasized, and presented shapes audience interpretation and response. In the context of e-cigarette prevention, this suggests that various videos’ content frames may differentially impact adolescent receptivity and behavior change intentions.

This study aims to explore Chinese middle-school students’ perspectives on e-cigarette prevention video characteristics. Our primary research questions were: 1) ‘Which characteristics of e-cigarette prevention videos do Chinese adolescents perceive as effective in discouraging e-cigarette use?’; 2) ‘Which characteristics are perceived as ineffective or counterproductive?’; and 3) What recommendations do adolescents offer for improving prevention video design? By centering adolescent voices in identifying effective prevention strategies, this research aims to inform the development of culturally appropriate and developmentally suitable e-cigarette prevention materials for Chinese youth.

## METHODS

### Study design and theoretical framework

This qualitative study employed focus group methodology with thematic analysis to explore adolescents’ perspectives on e-cigarette prevention video effectiveness, conducted in Beijing and Kunming, China, during March 2021. We selected this approach based on its demonstrated effectiveness in capturing adolescent viewpoints on health messaging and its capacity to generate rich, contextual data about complex phenomena^10^. The study design was informed by frame theory, which provided a theoretical lens for understanding how different message characteristics influence adolescent interpretation and response to prevention content.

### Research team and reflexivity

The research team consisted of seven members with backgrounds in public health, health communication, and adolescent psychology. The principal investigator had experience in tobacco control research and qualitative methods. Two trained moderators, both fluent in Mandarin and experienced in adolescent research, conducted the focus groups. To address potential researcher bias, we maintained reflexive journals throughout data collection and analysis, regularly discussing our assumptions about e-cigarette prevention and adolescent behavior. None of the researchers had prior relationships with participants, minimizing social desirability bias. As adult researchers studying adolescent perspectives, we sought to minimize bias by fostering a neutral and open environment for participants’ authentic expression.

### Setting and sampling strategy

We employed purposive sampling to select participants from two Chinese cities with contrasting tobacco control environments: Beijing, which has implemented comprehensive smoke-free legislation aligned with WHO Framework Convention on Tobacco Control standards, and Kunming, located in China’s primary tobacco-growing region with historically lower tobacco control awareness^11^. This sampling strategy aimed to capture diverse perspectives potentially influenced by varying regulatory contexts.

Within each city, we selected one middle school through consultation with local education authorities. Schools were chosen based on their willingness to participate. We selected one middle school each in Beijing and Kunming and, within each school, conducted one gender-stratified focus group (boys and girls), yielding four homogeneous groups (about 10 participants per group) to capture key heterogeneity along the city-context × gender strata. For sample size justification, Guest et al.^12^ empirically demonstrated that 2–3 focus groups identify ≥80% of themes, whereas 3–6 groups capture approximately 90%, with the most prevalent themes typically emerging within the first three groups. Aligned with our objective – to identify the principal, actionable message features of prevention videos – this four-group design was sufficient to provide robust evidence for core themes across contexts.

### Participant recruitment and eligibility

Recruitment occurred through school administrators who distributed study information to all students aged 13–15 years. Eligibility criteria included: 1) current enrollment in participating schools; 2) aged 13–15 years; 3) no current or former e-cigarette use; 4) ability to provide assent and obtain parental consent; and 5) fluency in Mandarin Chinese. We excluded current or former e-cigarette users to focus on prevention rather than cessation perspectives.

### Ethical considerations

The study received ethical approval from The Institutional Review Board of Peking University (IRB00001052-20056). We obtained written informed consent from parents/guardians and written assent from all participants. Participants were assured of confidentiality, voluntary participation, and the right to withdraw at any time without consequences. We provided age-appropriate information about the study purpose and procedures, emphasizing that there were no right or wrong answers and that honest opinions were valued. Each participant received a small educational gift worth approximately 20 RMB (about US$2.8) as appreciation for their time.

### Stimulus materials

We selected four international e-cigarette prevention videos as stimulus materials, chosen to represent diverse approaches to prevention messaging (Table 1). Selection criteria included variation in: 1) Message type (fear appeal vs rational persuasion); 2) Narrative style (personal stories vs expert information); 3) Visual approach (live action vs animation); and 4) Duration (30 seconds to 4 minutes). Videos were professionally translated and subtitled in simplified Chinese, with cultural adaptation limited to language to preserve original messaging approaches. Video characteristics were analyzed by the research team using a standardized coding framework.

**Table 1:** Characteristics of stimulus videos used in focus group discussions

*Video title*	*Type*	*Duration*	*Message frame*	*Visual style*	*Key content elements*
‘Whose Brain Is This?’	Public service announcement	30 s	Fear appeal	Live action with special effects	Metaphor of e-cigarettes as plague; emphasis on brain damage, formaldehyde exposure, irreversible lung damage
‘Dangers of Vaping’	Educational advertisement	1 min 40 s	Rational persuasion	Animation with expert narration	Marketing tactics exposure; nicotine addiction; harmful ingredients like diacetyl; adolescent brain development impacts
‘Ontario Teen’s Vaping Illness’	News report	2 min 13 s	Personal narrative	Documentary style	Real case of Kyle Boyd’s death; mother’s testimony; physician explanations of severe lung damage; policy responses
‘Doctor’s Stern Warning’	News feature	4 min 34 s	Medical authority	News format with interviews	Adam’s hospitalization story; expert analysis of JUUL marketing; lawyer’s perspective; long-term health impacts

### Data collection procedures

Participants were openly recruited within the campus. Participant demographic information including age was collected through a brief questionnaire. Focus groups were conducted in quiet rooms at participating schools. Each session followed a semi-structured discussion guide developed through literature review and pilot testing^6,13^. Groups were stratified by gender (male, female) and city (Beijing, Kunming), resulting in four groups of 10 participants each.

Sessions began with introductions and rapport-building activities appropriate for adolescents. Participants then viewed all four videos, with each shown twice to ensure comprehension. The viewing order was randomized across groups to minimize order effects. Following video viewing, trained moderators facilitated discussions using open-ended questions exploring: 1) immediate reactions and feelings; 2) perceived effective elements and reasons; 3) perceived ineffective elements and reasons; and 4) suggestions for improvement.

Moderators employed age-appropriate facilitation techniques, including think-pair-share activities and anonymous written responses for sensitive topics. We explicitly defined ‘effective’ as video elements that might discourage e-cigarette use and ‘ineffective’ as elements unlikely to prevent use or potentially counterproductive. Discussions lasted 40–90 minutes and were audio-recorded with participant permission. A research assistant took detailed field notes capturing non-verbal communication and group dynamics.

### Data analysis

We employed Braun and Clarke’s six-phase reflexive thematic analysis approach, recognized as particularly suitable for exploring patterns in qualitative data while maintaining theoretical flexibility^14^. Analysis proceeded through the following phases:

- Phase 1 - Familiarization: All recordings were professionally transcribed verbatim in Chinese. The research team read transcripts multiple times while listening to recordings, noting initial observations about content and emotional expression.
- Phase 2 - Generating initial codes: Two researchers independently coded the first two transcripts using inductive coding, identifying meaningful segments related to video effectiveness. The team met to compare codes, resolve discrepancies, and develop a preliminary coding framework.
- Phase 3 - Searching for themes: Codes were collated and organized into potential themes using NVivo 12 software. We examined relationships between codes, grouping them into broader patterns of meaning related to effectiveness and ineffectiveness. Data saturation was assessed by monitoring the emergence of new codes and themes; saturation was achieved when no new substantial themes emerged from the final focus group transcripts.
- Phase 4 - Reviewing themes: Candidate themes were reviewed against coded extracts and the full dataset. We ensured themes were internally coherent and distinctly bounded. Themes that lacked sufficient data support were refined or discarded.
- Phase 5 - Defining and naming themes: Each theme was clearly defined with a detailed description of its scope and relevance to research questions. A thematic map was developed illustrating relationships between themes, subthemes, and sub-subthemes.
- Phase 6 - Producing the report: Representative quotations were selected to illustrate each theme, balancing voices across gender and city. We ensured quotations were contextualized and clearly linked to analytic claims.

### Trustworthiness and rigor

We employed multiple strategies to enhance trustworthiness. Credibility was established through prolonged engagement with data, peer debriefing sessions, and member checking where preliminary findings were shared with a subset of participants. Dependability was ensured through detailed audit trails documenting analytical decisions. Confirmability was addressed through researcher triangulation, with multiple team members independently analyzing portions of data. Transferability was enhanced through thick descriptions of context and participant characteristics.

## RESULTS

### Participant characteristics

The study included 40 participants evenly distributed across Beijing (n=20) and Kunming (n=20), with equal gender representation (50% male, 50% female). All participants were aged 13–15 years and enrolled in local middle schools. None reported current or previous e-cigarette use, though several mentioned exposure to e-cigarette marketing or peers who used e-cigarettes (Table 2).

**Table 2:** Participant demographics and distribution, Beijing and Kunming, China, March 2021 (N=40)

*Characteristics*	*n (%)*
**Gender**	
Male	20 (50.0)
Female	20 (50.0)
**City**	
Beijing	20 (50.0)
Kunming	20 (50.0)
**Age** (years)	
13	12 (30.0)
14	18 (45.0)
15	10 (25.0)
**Focus group distribution**	
Beijing male	10 (25.0)
Beijing female	10 (25.0)
Kunming male	10 (25.0)
Kunming female	10 (25.0)

### Thematic analysis results

Analysis identified two overarching domains – effective and ineffective video characteristics – each containing multiple themes and subthemes. Table 3 presents the complete thematic structure.

**Table 3:** Thematic structure of adolescent perceptions of e-cigarette prevention video characteristics, Beijing and Kunming, China, March 2021 (N=40)

*Domain*	*Theme*	*Subtheme*	*Description*
**Effective characteristics**	Content and messaging	Real cases and testimonials	Authentic stories of actual victims increase credibility and emotional impact
		Specific health consequences	Concrete, visualized damage to organs creates tangible risk perception
		Chemical component disclosure	Scientific information about harmful substances enhances risk awareness
		Disturbing but memorable imagery	Appropriately shocking visuals create lasting impressions
	Style and presentation	Brief duration	Videos under 3 minutes maintain attention and message retention
		Visual impact and details	High-quality graphics showing bodily effects enhance message power
		Fear appeals	Moderate fear arousal motivates prevention without causing reactance
		Emotional narratives	Personal stories create empathy and identification
**Ineffective characteristics**	Content and messaging	Low information acceptance	Complex terminology and abstract concepts reduce comprehension
		Poor perceived effectiveness	Content fails to create memorable impressions or behavior change motivation
		Vague harm presentation	Non-specific warnings lack credibility and personal relevance
		Negative emotional responses	Excessive fear or disgust triggers avoidance rather than engagement
	Style and presentation	Animation format	Cartoon style perceived as childish and lacking authenticity
		Didactic expert lectures	Authoritative tone creates psychological distance and skepticism
		Excessive length	Videos over 2 minutes lose adolescent attention and interest

### Effective video characteristics

*Theme 1: Content and messaging features*

Participants consistently identified authentic, specific, and scientifically grounded content as most effective in discouraging e-cigarette use. Real cases emerged as particularly powerful, with participants emphasizing how genuine stories of young victims created personal relevance:

*‘This video uses real examples made into a film, showing someone who couldn’t walk, lying in bed, with detailed presentations of bodily harm. This warned us strongly about smoking’s major dangers, helping us understand the importance of not vaping*.*’* (Male, Kunming)

*‘The real case where he already died—this kind of example can better warn people, because his e-cigarette use behavior was truly dangerous.’* (Female, Kunming)

Specific visualization of health consequences resonated strongly across groups. Participants valued concrete depictions of organ damage over abstract warnings:

*‘That advertisement might be more effective the more uncomfortable it makes people feel. After seeing this, they might think about various bodily reactions from vaping, possibly raising their health consciousness*.*’* (Male, Kunming)

*‘The image of bugs crawling on skin and veins bulging gave me direct impact, making me feel the harm is severe.’* (Female, Beijing)

Scientific information about chemical components provided cognitive reinforcement for emotional responses:

*‘Showing smoking’s dangers and explaining what toxic substances are in e-cigarettes, how those substances harm us, more directly reflects e-cigarette dangers*.*’* (Male, Kunming)

*Theme 2: Style and presentation features*

Stylistic elements significantly influenced perceived effectiveness. Duration emerged as critical, with participants preferring concise messaging:

*‘Within acceptable range, three minutes or less allows relatively complete, focused viewing.’* (Male, Kunming)

*‘Thirty to forty seconds, one-minute videos can be watched quickly, and the content is easier to remember.’* (Female, Kunming)

Fear appeals, when appropriately calibrated, proved highly effective:

*‘This kind of direct visual impact is relatively large, making us better feel its harmfulness.’* (Female, Beijing)

Emotional narratives complementing factual information enhanced engagement:

*‘Expert narration format should be reduced. Rather scary-looking content with stories adapted from real events would be better.’* (Male, Beijing)

### Ineffective video characteristics

*Theme 3: Content and messaging limitations*

Participants identified several content-related factors that reduced effectiveness. Low information acceptance occurred when content was overly complex:

*‘After all, for someone like me who’s a poor student, seeing scientific educational videos using knowledge points to teach professional things, I simply can’t watch.’* (Female, Beijing)

Poor memorability undermined potential impact:

*‘Experts said many specific things. But after watching, I didn’t remember a single sentence.’* (Female, Kunming)

*‘The video felt very boring and long, then after watching there was no impression.’* (Female, Beijing)

Vague harm presentation failed to create perceived vulnerability:

*‘I think the harm presentation wasn’t specific enough. The video’s bugs entering the body made me think of bacteria, many people wouldn’t connect it to cigarettes.’* (Female, Kunming)

*‘This video just tells you theory not to smoke, but if you really want to smoke it’s useless. Just tells you it’s best not to smoke, then mentions consequences. The consequences lack impact, just telling you might get sick, but probability is small, so many people still smoke.’* (Female, Beijing)

*Theme 4: Style and presentation barriers*

Animation format consistently received negative responses:

*‘This kind of animation doesn’t have the persuasive power of real cases. I don’t really like this animated style, gives feeling of weak persuasiveness.’* (Female, Kunming)

Didactic expert presentations created psychological distance:

*‘Some experts talking about what harms it causes feels condescending. After all, there are many socalled experts now, somewhat unbelievable.’* (Female, Kunming)

Excessive length led to disengagement:

*‘This video is like those subway advertisements—too long, so they don’t go viral, or no one has patience to watch.’* (Male, Kunming)

*‘Such long videos can’t grasp the key points. If I see videos with long progress bars, if it’s not my favorite type, I just skip directly*.*’* (Male, Kunming)

### Recommendations for future videos

Participants offered specific suggestions for optimizing prevention video design. Content recommendations emphasized authentic narratives with concrete consequences:

*‘Reduce expert narration format. Prefer watching visuals rather than hearing too much preaching language or seeing too much text*.*’* (Male, Beijing)

*‘Adding more real smokers’ examination cases showing harm would be better. Would make me feel it has specific authenticity.’* (Female, Kunming)

*‘Include specific harms, like possibly causing death—concrete consequences should be specifically reflected.’* (Male, Kunming)

Style recommendations focused on balanced fear appeals with brief duration:

*‘For widespread dissemination, video time must be short, but cover comprehensive content*.*’* (Male, Kunming)

*‘Could be improved by being slightly shorter. Still feels a bit long, hard to concentrate*.*’* (Male, Beijing)

*‘More terrifying harms from smoking, telling me scary consequences. After watching, definitely wouldn’t want to try.’* (Male, Kunming)

## DISCUSSION

This study provides valuable insights into Chinese adolescents’ perspectives on e-cigarette prevention video effectiveness, revealing clear preferences for authentic, specific, and emotionally engaging content over animated or didactic approaches. Our findings contribute to the growing body of literature on culturally appropriate tobacco prevention strategies while addressing the specific developmental needs of early adolescents.

### Theoretical and practical implications

Our results strongly support frame theory’s relevance to health communication with adolescents. The effectiveness of real cases and specific health consequences aligns with episodic framing, which uses concrete examples rather than abstract statistics to enhance message relevance^15^. Chinese adolescents’ preference for episodic over thematic framing may reflect both developmental factors – concrete operational thinking predominating in early adolescence – and cultural factors emphasizing collective learning through cautionary examples.

The finding that fear appeals combined with factual information proved most effective corroborates recent meta-analyses showing moderate fear arousal with high efficacy messages optimizes prevention outcomes^16^. However, our participants’ emphasis on ‘appropriate’ fear levels and rejection of excessive disturbing content, highlights the delicate balance required. This aligns with the Extended Parallel Process Model, which predicts that excessive fear without perceived efficacy triggers defensive avoidance rather than protective behavior^17^.

Participants’ strong rejection of animation contradicts some Western research suggesting animated content effectively engages youth^18^. This cultural difference may reflect Chinese adolescents’ association of animation with entertainment rather than serious health information, or developmental desires to be treated as mature individuals rather than children^13^. The rejection of didactic expert testimonials similarly suggests that traditional authority-based messaging may be less effective with contemporary Chinese youth, possibly reflecting broader generational shifts in information source credibility.

### Contextualizing findings within Chinese policy environment

Our findings gain particular significance given China’s evolving e-cigarette regulatory landscape. Despite comprehensive regulations implemented in 2022^1,3,19^, including flavor bans and enhanced age verification requirements, recent studies document widespread non-compliance, with nearly half of underage purchases involving no age checks^5^. This enforcement gap heightens the importance of effective prevention education as a complementary strategy.

The preference for real cases showing severe consequences may partly reflect that graphic cautionary tales may serve as informal risk communication, when formal protective systems prove inadequate. This interpretation is supported by participants’ emphasis on ‘truly dangerous’ outcomes and death as ultimate consequences – suggesting they seek unambiguous risk information to counter mixed messages from continued e-cigarette marketing, despite official restrictions.

### International comparisons and cultural considerations

Comparing our findings with previous qualitative research reveals both universal and culturally specific patterns. The preference for brief videos (under 3 minutes) aligns with global trends in adolescent media consumption and shortened attention spans in digital environments^13^. However, Chinese adolescents’ specific rejection of Western-style animation and preference for documentary-style real cases suggests cultural variation in perceived credibility markers.

Recent qualitative studies from the United States and Europe report adolescents responding positively to peer-led messaging and social media influencer content^6,20^. While our participants did not explicitly discuss peer messengers, their emphasis on ‘real’ young victims suggests potential receptivity to peer testimonials, provided they convey authentic experiences rather than scripted messages.

### Implications for prevention video development

Our findings provide insight for developing culturally appropriate e-cigarette prevention videos for Chinese adolescents. First, prioritize authentic narratives featuring real adolescents or young adults who experienced e-cigarette-related health consequences. These stories should include specific medical details and visual evidence of harm, presented through documentary-style filming rather than animation or purely statistical presentations.

Second, respect adolescents’ time and attention constraints by limiting videos to 1–3 minutes while maintaining comprehensive content^16^. This requires careful message prioritization, focusing on most impactful elements: personal narrative hook, specific health consequences, chemical dangers, and brief empowerment message. Longer form content might be segmented into series of brief videos for sequential viewing.

### Strengths and limitations

This study’s strengths include its qualitative depth, sampling across two distinct cities, gender balance, and focus on the understudied early adolescent population. Using international stimulus videos allowed exploration of diverse messaging approaches, while focus group methodology captured peer interaction effects relevant to social influence processes in e-cigarette initiation.

However, several limitations warrant consideration. First, excluding current e-cigarette users focused findings on prevention rather than cessation, potentially missing insights from adolescents who may have different perspectives on prevention message effectiveness. Second, using translated Western videos may have introduced a cultural disconnection beyond what language adaptation could address. Third, social desirability bias may have influenced participants’ reported responses, particularly given school-based recruitment and adult moderation. Fourth, the convenience sampling of schools based on willingness to participate may have introduced selection bias. Additionally, our sample from two cities, while providing contrasting tobacco control contexts limits the generalizability of findings to China’s full geographical and socioeconomic diversity. Rural adolescents, ethnic minorities, and youth not attending mainstream schools may have different perspectives requiring additional research. Finally, as a qualitative study, these findings cannot establish causal relationships between video characteristics and actual prevention behaviors.

### Future research directions

This study identifies several priorities for future research. Experimental studies should test effectiveness of videos incorporating our identified characteristics using behavioral outcomes rather than solely perceptual measures. Longitudinal research could examine whether initial positive responses to fear appeals maintain effectiveness or diminish through habituation.

Cultural adaptation research should explore optimal strategies for translating effective prevention approaches across cultural contexts while maintaining core effective elements. Given rapid changes in e-cigarette products and marketing, studies should examine how prevention messages can remain relevant as industry tactics evolve.

Finally, implementation research should investigate optimal dissemination channels for prevention videos, particularly through social media platforms where adolescents encounter pro-vaping content. Understanding how to position prevention messages within adolescents’ digital media environments may prove as important as message content itself.

## CONCLUSIONS

This qualitative study provides essential insights into Chinese adolescents’ perspectives on e-cigarette prevention video effectiveness, revealing clear preferences for authentic, specific, and emotionally engaging content delivered through brief, high-impact formats. The identification of real cases and fear appeals as particularly effective, contrasted with rejection of animation and didactic approaches, offers concrete guidance for developing culturally appropriate prevention materials.

These findings provide evidence-based guidance for developing e-cigarette prevention videos for Chinese adolescents. The preference for authentic narratives and specific health consequences suggests that effective prevention materials should prioritize documentary-style content featuring real experiences over animated or didactic approaches. Further research is needed to test the effectiveness of videos incorporating these characteristics and to determine optimal dissemination strategies for reaching target populations.

## Data Availability

The data supporting this research are available from the authors on reasonable request.
